# Outpatient anti-spike monoclonal antibody administration is associated with decreased morbidity and mortality among patients with cancer and COVID-19

**DOI:** 10.21203/rs.3.rs-2433445/v1

**Published:** 2023-01-09

**Authors:** Panos Arvanitis, Alexis Hope Lerner, Kendra Vieira, Nouf Almaghlouth, Dimitrios Farmakiotis

**Affiliations:** Division of Infectious Diseases, Warren Alpert Medical School of Brown University; Division of Infectious Diseases, Warren Alpert Medical School of Brown University; Division of Infectious Diseases, Warren Alpert Medical School of Brown University; Division of Infectious Diseases, Warren Alpert Medical School of Brown University; Division of Infectious Diseases, Warren Alpert Medical School of Brown University

**Keywords:** COVID-19, SARS-CoV-2, cancer, infection, anti-spike monoclonal antibodies

## Abstract

**Background:**

Patients with cancer have many comorbidities that increase their risk of death from Coronavirus disease 2019 (COVID-19). Anti-spike monoclonal antibodies (mAbs) reduce the risk of hospitalization or death from COVID-19 in the general population. To our knowledge, no studies have focused on the clinical efficacy of mAbs compared to no outpatient treatment exclusively among patients with solid tumors and hematologic malignancies, who are often excluded from clinical trials.

**Methods:**

We studied patients with cancer who had COVID-19 between 11.9.2020 and 7.21.2022 and received mAbs in an outpatient setting. We compared hospitalization and mortality rates to those of patients with cancer concurrently diagnosed with COVID-19, who were eligible for mAbs, but did not receive any outpatient treatment.

**Results:**

63 patients received mAbs and 89 no outpatient treatment. Administration of mAbs was associated with lower 90-day hospitalization (20.6% vs. 60.7%, p<0.001), all-cause (6.3% vs. 19.1%, p=0.025) and COVID-19-attributed (3.2% vs. 14.6%, p=0.019) mortality rates, and lower peak O2 requirements (ordinal Odds Ratio [OR]=0.33, 95%Confidence Intervals [CI]=0.20–0.53). Administration of mAbs (aHR 0.21, p<0.001), age (≥ 60 years, adjusted Hazard Ratio [aHR] 1.86, p=0.033), and metastases (aHR 0.41, p=0.007) were independently associated with hospitalization. mAb treatment remained significantly associated with all-cause (aHR 0.27, p=0.019) and COVID-19-attributed (aHR 0.19, p=0.031) mortality, after adjustment for other factors.

**Conclusions:**

mAb administration was associated with improved clinical outcomes among vulnerable patients with cancer and COVID-19. With no mAbs approved currently for treatment against the prevalent circulating variants, the development of new mAbs should be a research priority.

## Introduction

Patients with cancer are a heterogeneous group with an overall increased risk of hospitalization and death when infected with SARS-CoV-2^1^. Patients with active or prior malignancies often experience compounding clinical risk factors for severe COVID-19, such as older age, multiple comorbidities, immunosuppressive treatment, metastatic disease, and high contact rates with healthcare services that may increase their risk of contracting COVID-19^1^. Furthermore, patients with cancer—especially hematologic malignancies (HM)—tend to mount a weaker immune response to COVID-19 vaccination than their immunocompetent peers^2–4^.

Several clinical trials^5–7^ and observational studies^8, 9^ have established the protective role of anti-spike monoclonal antibodies (mAbs) in reducing the severity of clinical outcomes among eligible outpatients. However, cancer-related data in these studies were relatively opaque, given that the investigators frequently: (1) pooled patients with cancer and patients who had other immunocompromising conditions, such as organ transplant recipients (OTR)^8^; and (2) did not provide information on specific cancer characteristics such as type of cancer or anti-neoplastic treatment modalities^10^.

To our knowledge, no study has previously assessed the efficacy of mAbs in preventing hospitalization and death exclusively among patients with solid and hematologic malignancies, compared to contemporary controls. Using data from our comprehensive institutional registry, we retrospectively evaluated clinical outcomes following outpatient-administered anti-spike mAb therapy to patients with solid or hematologic cancers and COVID-19, compared to those of patients with cancer who were eligible to receive mAbs but did not.

## Methods

### Study Design and Data Collection

We retrospectively studied patients with history or active cancer at Brown University-affiliated hospitals, diagnosed with SARS-CoV-2 infection between November 9, 2020 (date of Emergency Use Authorization [EUA] for the first mAb-bamlanivimab), and July 21, 2022. Patients were excluded if they met any of the following criteria: (1)<18 years old; (2) received nirmatrelvir/ritonavir (Paxlovid^®^); were not eligible for mAbs under EUA, specifically: (3) hospitalized for COVID-19 at presentation, even if they received mAbs while inpatient; (4) had symptoms for more than 10 days; or (5) presented with high O_2_ requirements due to COVID-19, compared to baseline^11, 12^. The study was approved by the Lifespan Institutional Review Board.

The primary outcome was COVID-19-related hospital admission within 90-days after diagnosis (the date of the positive test). Secondary outcomes were survival rates (90-day all-cause or COVID-19-attributed mortality [after exclusion of patients who died from other reasons]), length of hospital stay, and peak (worst) O_2_ requirements on a modified ordinal scale as follows: 0, outpatient only; 1, admitted to the hospital but without supplemental O_2_ requirement; 2, low-flow O_2_ requirement; 3, high-flow O_2_ requirement; 4, non-invasive mechanical ventilation (Bilevel Positive Airway Pressure (BiPAP), continuous positive airway pressure (CPAP)); 5, invasive mechanical ventilation.

For survival analyses, the follow-up time of 90 days was chosen because patients with cancer have comorbidities and diverse baseline characteristics which weigh more heavily on mortality later in disease progression^13^. For COVID-19-attributed mortality, we excluded patients who died within 90 days from reasons other than COVID-19, instead of considering such death as competing event, given the potential complex effect of cancer prognosis on the decision to treat COVID-19 with mAbs.

### Statistical Analyses

The normality of distribution was assessed with the Kolmogorov-Smirnoff test. Continuous variables are presented as medians (Interquartile Range [IQR]), while nominal and ordinal variables as numbers (%). The differences between the two groups were compared using Mann-Whitney U-criterion, Fisher’s exact or Mantel-Haenszel tests, respectively.

The 90-day survival was assessed by Kaplan-Meier curves, log-rank test, univariable and multivariable Cox regression models. For Cox regression analysis, we excluded variables, if >20% of data were missing. The proportional hazards assumption was confirmed by a visual assessment of Schoenfeld residuals. Factors with a *p*-value of <0.1 on univariable analyses were entered in the multivariable models. (Adjusted) Hazard Ratios (aHR) along with 95% Confidence Intervals (CI) are reported.

The association between mAb administration and peak O_2_ requirements was assessed by ordinal logistic regression analysis and the proportional odds assumption was tested with the Score test. Odds ratios (OR) along 95%CI are reported.

All analyses were performed with R, version 4.0.5 (R Foundation for Statistical Computing). Statistical significance was set at a two-tailed *p*-value of 0.05, unless otherwise indicated above.

## Results

### Baseline Demographic and Clinical Characteristics

**Figure 1:**
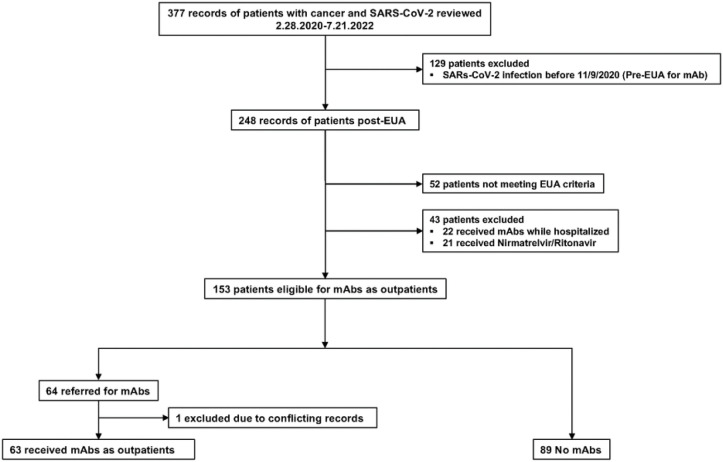
Patient Selection Flow diagram illustrating patient selection. mAbs=Anti-spike monoclonal antibodies, EUA=Emergency Use Authorization

During the study period, two hundred forty-eight patients at our center contracted SARS-CoV-2. Of those, one hundred and fifty-three met the criteria for outpatient mAb administration, after exclusion of patients treated with nirmatrelvir/ritonavir (Paxlovid^®^). Sixty-three received mAb, and eighty-nine did not (Figure 1). One patient was referred for mAb infusion, but it was unclear by chart review if they received it, therefore that patient was excluded. 27% (17/63) received bamlanivimab, and 9.5% (6/63) received sotrovimab. The majority of patients (61.9%, 39/63), received the combination of either bamlanivimab/etesevimab or casirivimab/imdevimab, depending on availability at the infusion clinic. One patient (1.6%) received bebtelovimab.

Baseline demographic and clinical characteristics of these two groups were largely comparable (Table 1). 51% (78/152) of patients identified as male, the median age was 67 (IQR 55–75) years, and 52% (79/152) of the patients were current or former smokers. Most patients contracted COVID-19 in 2021 and 2022, while only 14.5% (22/152) in late 2020, most of whom (18) did not receive mAbs. There were no significant differences in vaccination status and the number of doses between groups.

The distribution of cancer characteristics between the two groups is summarized in Table 2 The majority of patients had solid tumors (69.7%, 106/152). ECOG scores were not reported for 31 patients (7 who received mAbs, 24 who did not). The most common anticancer treatment amongst patients who received mAbs was cytotoxic therapy (61.9%, 39/63), and locoregional for the non-mAb cohort (50/89, 56.2%). Patients who received mAbs were more likely than their counterparts to have received cytotoxic therapy (61.9% vs. 42.7%, *p*=0.020).

### Clinical Outcomes

**Figure 2:**
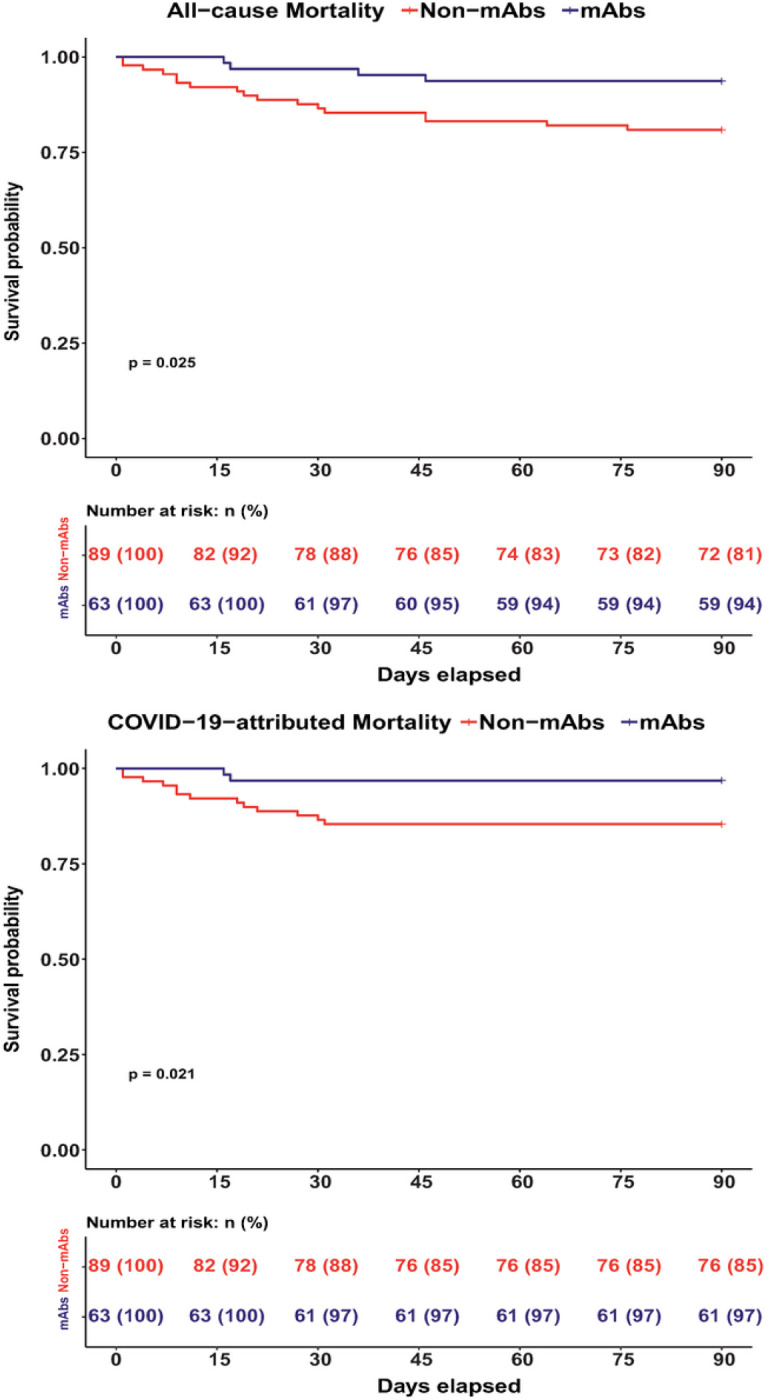
Kaplan-Meier Survival Curves Kaplan–Meier 90-day All-cause Mortality and COVID-19 attributed mortality curves for patients who received anti-spike monoclonal antibodies for the treatment of SARS-CoV-2 infection (mAbs) and those who did not (Non-mAbs).

**Figure 3:**
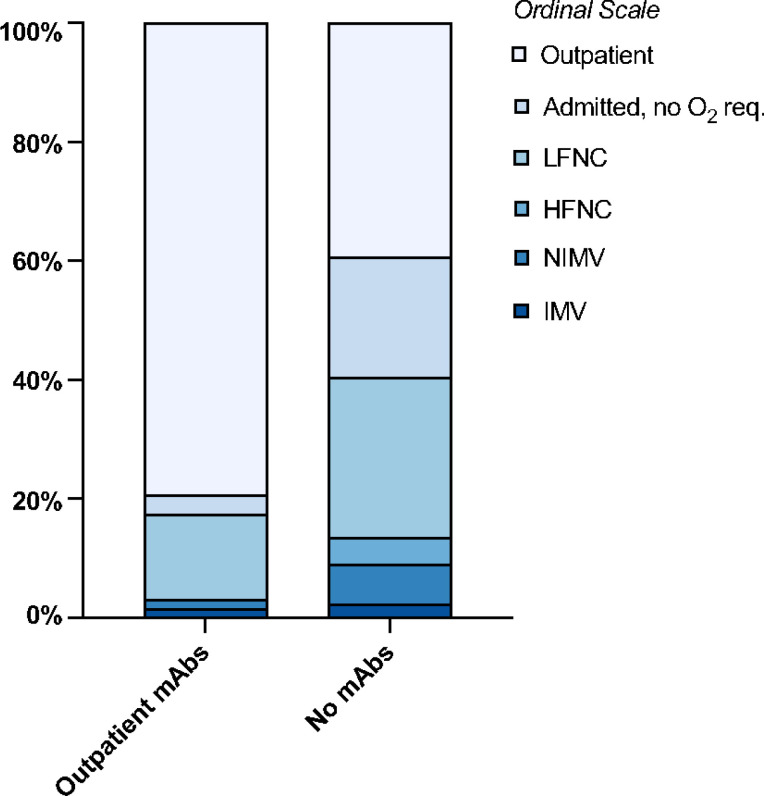
Peak O_2_ requirement ordinal scale value distribution by mAb status LFNC= low-flow nasal cannula; HFNC= high-flow nasal cannula; 4, NIMV=non-invasive mechanical ventilation (BiPAP, CPAP); IMV= invasive mechanical ventilation

Clinical outcomes are summarized in Table 3 and Kaplan-Meier survival curves are shown in Figure 2. Of 63 patients who received mAbs, 13 (20.6%) were hospitalized, strikingly less than the 54 of 89 patients (60.7%) who did not receive mAbs (*p*<0.001). Patients who received mAbs had lower rates of 90-day all-cause (6.3% vs. 19.1%, *p*=0.025), and COVID-19-attributed mortality (3.2% vs. 14.6%, *p*=0.019). Differences in length of hospital stay were not significant. Patients who did not receive mAbs were 3.03 times more likely to have higher peak O_2_ requirements than patients who received mAbs (ordinal OR=0.33, 95% CI=0.20–0.53) (Figure 3).

### Multivariable Analyses

**Figure 4:**
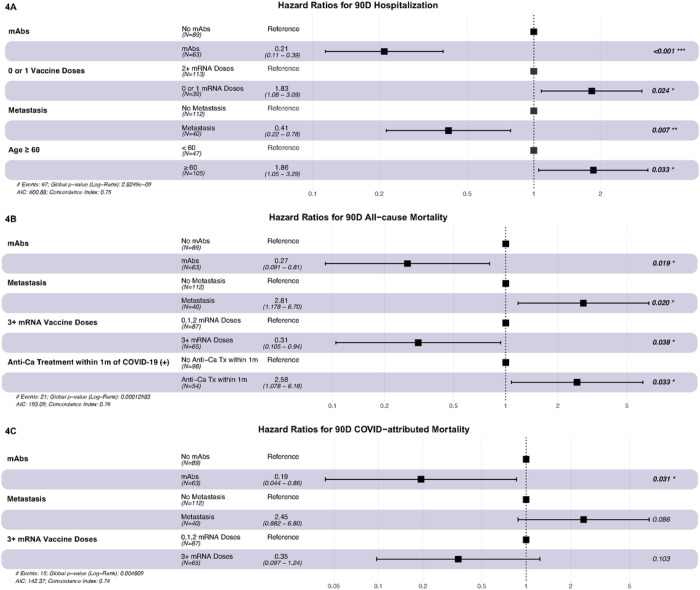
aHR for 90-day hospitalization, all-cause mortality and COVID-19-related mortality Multivariable Cox regression model for primary outcome (COVID-19-related hospital admission within 90-days after positive test) and secondary outcomes (90 Day All-cause Mortality and COVID-19 attributed mortality). mAbs= anti-spike monoclonal antibodies

On Cox regression analysis, independent risk factors for 90-day hospitalization were vaccination status (unvaccinated or after 1 vs. >1 mRNA vaccine doses), metastatic disease, and age ≥ 60. Adjusting for the aforementioned factors, mAb administration was still associated with decreased risk of hospitalization by day 90 (adjusted hazard ratio [aHR]=0.21, *p*<0.001) (Figure 4A).

Independent risk factors for 90-day all-cause mortality ^³^3 vs. <3 mRNA vaccine doses, and anticancer treatment less than 1 month before testing positive for COVID-19. Adjusting for the factors above, mAb administration was still associated with increased 90-day survival (aHR=0.27, *p*=0.019) (Figure 4B).

Independent risk factors for 90-day COVID-19-attributed mortality were metastatic disease and vaccination with ^³^3 vs. <3 mRNA vaccine doses. Adjusting for these factors, mAb administration was associated with increased 90-day survival (aHR=0.19, *p*=0.031) (Figure 4C).

## Discussion

Patients with cancer who contract COVID-19 are more vulnerable than the general population at every stage of the COVID-19 continuum: from contagion exposure to breakthrough COVID-19 after vaccination, hospitalization, critical illness, prolonged morbidity (Post-Acute Sequelae of COVID [PASC]/“long-COVID”) and death^3, 4, 10^. Kuderer et al.^1^ previously studied a cohort of 900 clinically and demographically diverse patients with cancer from the COVID-19 and Cancer Consortium (CCC19) registry. Several cancer-specific (worse ECOG status and active malignancy) and non-cancer-specific (male sex, older age, positive smoking history, number of comorbidities, and receiving hydroxychloroquine and azithromycin) parameters were associated with increased 30-day all-cause mortality.

Many non-cancer-specific features (e.g., older age) are more common among individuals with cancer. Moreover, people with cancer may experience immune suppression from the state of malignancy itself or medication-related such as antineoplastic therapy and steroids^1, 14^, leading to a decreased humoral response to vaccination^15, 16^, increased risk for breakthrough infection^4, 17, 18^, and worse overall clinical outcomes from COVID-19^19^. Patients with cancer also have more frequent and prolonged healthcare interactions compared to their peers without chronic or disabling illness, due to the extended temporal nature of antineoplastic treatment and follow-up with multiple providers, often across more than one healthcare settings, leading to increased risk of SARS-CoV-2 transmission^3^.

Importantly, COVID-19 complicates cancer care by limiting screening, diagnosis, and timely treatment options, potentially facilitating disease progression and significant psychological distress^3, 20^. For the above reasons, patients with cancer are a high-risk group that could benefit significantly from the timely initiation of effective treatment against SARS-CoV-2.

Monoclonal antibodies (mAbs) that block SARS-CoV-2 entry into host cells by binding to the viral spike glycoprotein have proven to be excellent outpatient therapeutic agents in clinical trials and quasi-experimental studies, when used against susceptible strains^5, 21, 22^. Since November 2020, six anti-SARS-CoV-2 mAbs (bamlanivimab, bamlanivimab/etesevimab, casirivimab/imdevimab, sotrovimab, bebtelovimab, and tixagevimab/cilgavimab [for primary prophylaxis]) – have received EUA, with only the last option still available, as the rest do not have activity against circulating Omicron variants any longer^12, 23^.

There is a relative paucity of data on the protective efficacy of mAbs specifically for patients with cancer^10^. In the phase 3 portion of the BLAZE-1 clinical trial that supported efficacy of bamlanivimab/etesevimab for patients with mild or moderate COVID-19^5^, patients with cancer were classified with other patients who have impaired immune system (e.g., solid organ transplant recipients), under the broad inclusion criterion of being immunocompromised, despite substantial, clinically-relevant variations in depth and types of immunosuppression. Similarly, Ganesh et al., in a study of more than 3,500 patients who received bamlanivimab or casirivimab/imdevimab, referred broadly to immunocompromised status, which is one of the inclusion criteria under the EUA^8^. In a retrospective cohort study by Jalbert et al.^9^ that included more than 13,000 patients who received casirivimab/imdevimab, patients with cancer or chemotherapy were included as a separate category for cohort-matching purposes, but their malignancy characteristics were not described, nor the direct effect of mAbs on this subpopulation. And in the COMET-ICE randomized clinical trial for sotrovimab, patients with cancer receiving immunosuppressive chemotherapy or immunotherapy were explicitly excluded^7^.

To the best of our knowledge, our study is the first to assess the efficacy of mAbs exclusively in COVID-19 patients with both solid and hematologic malignancies, compared to appropriate controls who did not receive mAbs or any other outpatient treatment, while adjusting for possible confounders. We observed a significant, sustained reduction in hospitalization rates and peak O_2_ requirements among sixty-three patients with cancer who received mAbs as outpatients, compared to eighty-nine who did not (Table 3, Figures 3 & 4). Additionally, patients with cancer and COVID-19 treated with mAbs had longer 90-day survival, compared to those who did not (Table 3, Figure 2). To our knowledge, such a mortality benefit from mAbs, compared to untreated patients, has not been previously shown in a cohort of patients with solid tumors and HM. Our findings agree with several other multicenter observational series of immunocompromised patients with mild or moderate COVID-19, who were given mAbs in the outpatient setting, which demonstrated lower than expected hospitalization and mortality rates^9, 22, 24^. Similarly, our results agree with the findings of a Czech multicenter study that included only patients with HM who received bamlanivimab or casirivimab/imdevimab^25^. In that cohort, the investigators found lower rate of progression to severe disease among patients with HM who received mAb compared to those who did not, and a borderline mortality benefit in the remdesivir/convalescent plasma “naïve” subgroup^25^.

Another important finding from our study was that vaccinated patients, especially those who had received ≥3 doses of an mRNA vaccine, had lower mortality rates (Figure 4), despite concerns for lower immunological vaccine efficacy among immunocompromised patients^15, 16^, and one small study from the CCC19 registry, which showed comparable clinical outcomes between unvaccinated patients with cancer and those who had received 2 doses of an mRNA vaccine^18^. The results of the present report are consistent with those of a previously published study at our center among organ transplant recipients^26^, and the updated CCC19 data^17^, highlighting that vaccination of immunocompromised patients, especially with additional “booster” doses, is an essential preventive strategy against severe COVID-19 and death.

Our study has limitations: First, data were retrospectively collected, but all outcome variables were clearly defined and easy to extract from the electronic medical record (EMR). Second, the single-center design may limit the generalizability of results. However, our findings are comparable with those from several larger multi-center studies and in agreement with the well-established benefits of mAbs in the general population. Third, whether an eligible patient receives mAbs is multifactorial and dependent on clinical judgement. ECOG status could play a role in these decisions, and we did not have enough entries to include it in our multivariable models. Fourth, the groups (mAbs vs. controls) were relatively small, and we did not perform propensity-score matching. Nonetheless, the treatment and non-mAbs groups had overall well-balanced baseline characteristics (Table 1) and the difference in clinical outcomes was significant even after appropriate multivariable adjustments.

Last, our findings no longer apply to circulating variants: Most patients in our study contracted SARS-CoV-2 during the peak of the Delta wave and at the beginning of the Omicron (BA.1 variant) wave; the majority of patients received bamlanivimab/etesevimab or casirivimab/imdevimab. In January 2022, the FDA limited the use of bamlanivimab/etesevimab and casirivimab/imdevimab for only non-Omicron variants^12^. After January 2022, >95% of SARS-CoV-2 infections in Rhode Island were caused by the Omicron variant, therefore these mAbs were no longer being administered in our State, reflecting the trend in the Northeastern US^27^. Likewise, the EUA for sotrovimab was retracted in April 2022, when the BA.2 Omicron sub-variant became dominant^12^. Bebtelovimab, which had EUA since February 2022 and was still active against most circulating Omicron variants, was underrepresented in our study, as only 1 patient received it. And in December 2022, the FDA revoked the EUA authorization for the last available COVID-19 monoclonal antibody treatment, bebtelovimab, as well. This was mainly justified by the lack of activity against Omicron subvariants BQ.1 and BQ.1.1, which at the time of this manuscript represent >60% of SARS-CoV-2 infections nationally^23^.

Thus, mAbs lost their clinical utility rather fast, as the result of spike protein mutations^28^ An equally effective oral antiviral against SARS-CoV-2, nirmatrelvir/ritonavir (Paxlovid^®^), has maintained its efficacy against all Omicron sub-variants^28^, and became the mainstay of outpatient (mild to moderate) COVID-19 treatment for many patients. Notwithstanding, ritonavir is a potent CYP inhibitor, and clinically relevant drug-drug interactions^29^ make its administration often challenging for patients on multiple medications, such as those with cancer.

In conclusion, we found that the administration of mAbs to non-hospitalized patients with cancer was associated with markedly decreased morbidity and mortality, compared to eligible for mAbs but untreated controls, after adjustment for possible confounders. Despite the wide availability of Paxlovid^®^, and based on the results of this study, we believe there is still an important role for passive immunization, e.g. high-titer convalescent plasma that has EUA for treatment of COVID-19 in immunosuppressed patients^30,31^. Moreover, the development of novel mAbs against emerging SARS-CoV-2 variants should be a research priority.

According to a recent position statement^10^, investigations and policies regarding ongoing or future pandemics should: (1) include patients with cancer in all treatment clinical trials, (2) collect specific data on cancer characteristics and treatment, and (3) include malignancy factors as covariates or as strata for subgroup analyses. We strongly support these suggestions, in addition to educating patients with cancer about treatments for COVID-19 that are available to them, to ensure timely access. Although cancer necessitates a close relationship between patients and health care providers that may facilitate iatrogenic exposure to COVID-19, with host and treatment factors predisposing to severe illness, the field of oncology also offers the opportunity for close, careful management in the setting of preexisting strong patient-provider alliances.

## Figures and Tables

**Table 1:** Baseline Characteristics

Parameter	Outpatient mAbs	No mAbs (controls)	*p-value*
Number of patients	63	89	
Age (years) (median-IQR)	67 (55.0–75.0)	68 (60–77.5)	*ns*
Male (%)	30 (47.6)	48 (53.9)	*ns*
BMI (kg/m^2^) (median-IQR)	27.75 (23.7–30.9)	26.90 (22.30–31.39)	*ns*
Race and ethnicity (%)			
*Hispanic*	11 (17.5)	10 (11.2)	*ns*
*Non-Hispanic Black*	4 (6.3)	8 (9.0)	*ns*
*Non-Hispanic White*	44 (69.8)	67 (75.3)	*ns*
*Other*	4 (6.3)	4 (4.5)	*ns*
Smoking status (%)			
*Never*	29 (46.0)	44 (49.4)	*ns*
*Current or former*	34 (54.0)	45 (50.6)	*ns*
Comorbid conditions^a^ (%)			
*Hypertension*	38 (60.3)	64 (71.9)	*ns*
*Diabetes*	26 (41.3)	38 (42.7)	*ns*
*Cardiac*	25 (39.7)	39 (43.8)	*ns*
*Renal*	4 (6.3)	23 (25.8)	*ns*
*Pulmonary*	21 (33.3)	32 (36.0)	*ns*
Received remdesivir (% of hospitalized)^b^	8 (12.7)	36 (66.7)	*ns*
Year of contracting SARS-CoV-2			
*2020*	4 (6.3)	18 (20.2)	**0.017**
*2021*	35 (55.6)	48 (53.9)	*ns*
*2022*	24 (38.1)	23 (25.8)	*ns*
mRNA vaccination status			
*Unvaccinated*	13 (20.6)	26 (29.2)	*ns*
*2 doses*	22 (34.9)	26 (29.2)	*ns*
*3+ doses*	28 (44.4)	37 (41.6)	*ns*
Anti-Spike mAb used (%)			
*Bamlanivimab*	17 (27.0)	N/A	N/A
*Sotrovimab*	6 (9.5)	N/A	N/A
*Bamlanivimab/etesevimab or casirivimab/imdevimab* ^c^	39 (61.9)	N/A	N/A
*Bebtelovimab*	1 (1.6)	N/A	N/A

Data are presented as number (percentage) for categorical variables and median (interquartile range [IQR]) for continuous variables. All patients were coded as either female or male in the EMR; none were listed as intersex. Ethnicity and race data were taken from the hospital

EMR and may not reflect patient self-identification. BMI=Body-Mass Index, IQR=interquartile range, mAb=anti-spike monoclonal antibody, ns=not significant (*P*>0.05).

a Total will be greater than the total number of patients due to row overlap.

b Hospital admission numbers are presented formally as a clinical outcome in Table 3. At our institution, remdesivir is exclusively reserved for hospitalized patients.

c The choice between bamlanivimab/etesevimab and casirivimab/imdevimab was based on local availability and could not be extracted from the EMR.

**Table 2:** Cancer Characteristics

Parameter	mAb	No mAb	*p*-value
Number of patients	63	89	
Solid tumors (%)	44 (69.8)	62 (69.7)	*ns*
Hematologic malignancy (%)	19 (30.2)	35 (39.3)	*ns*
Metastasis at time of COVID-19 diagnosis (%)	17 (27.0)	23 (25.8)	*ns*
ECOG Performance Status (%)			
*0*	16 (25.4)	20 (22.5)	*ns*
*1*	28 (44.4)	25 (28.1)	
*≥2*	12 (19.0)	20 (22.5)	
*Unknown*	7 (11.1)	24 (27.0)	**0.017**
Timing of most recent anticancer therapy prior to COVID-19 onset (%)			
*Not treated*	3 (4.8)	7 (7.9)	*ns*
*<1 month*	25 (39.7)	29 (32.6)	*ns*
*1–3 months*	9 (14.3)	8 (9.0)	*ns*
*>3 months*	26 (41.3)	45 (50.6)	*ns*
Anticancer therapy modality^a^ (%)			
*None*	3 (4.8)	7 (7.9)	*ns*
*Cytotoxic*	39 (61.9)	38 (42.7)	**0.020**
*Locoregional (surgery and/or radiation)*	33 (52.4)	50 (56.2)	*ns*
*Immunotherapy*	12 (19.0)	19 (21.3)	*ns*
*Targeted*	31 (49.2)	30 (33.7)	ns
*Endocrine*	4 (6.3)	10 (11.2)	*ns*
*Antimetabolite*	8 (12.7)	15 (16.9)	*ns*

Data are presented throughout as number (percentage). ECOG = Eastern Cooperative Oncology Group. ns = not significant (*p* > 0.05).

a Total will be greater than the total number of patients due to row overlap.

**Table 3:** Clinical Outcomes

Parameter	mAb	No mAb	*p*-value
Total number of patients	63	89	NA
Median time to mAb administration (days from symptom onset)	3 (2–7)	NA	NA
Hospital admission (n)	13 (20.6)	54 (60.7)	**<0.001**
Length of hospital stay (days) (median, IQR)	5 (2.5–9.5)	8 (4–14)	*ns*
90-day all-cause mortality (%)	4 (6.3)	17 (19.1)	**0.025**
90-day COVID-19-related mortality (%)	2 (3.2)	13 (14.6)	**0.019**

Data are presented as number (percentage) for categorical variables and median (interquartile range [IQR]) for continuous variables. mAb=monoclonal antibody, NA=Not Applicable.

## Data Availability

The data that support the findings of this study are available from the corresponding author, DF, upon reasonable request.

## Abbreviations

aHR: adjusted hazard ratio
BiPAP: Bilevel Positive Airway Pressure
COVID-19: Coronavirus disease 2019
CPAP: Continuous Positive Airway Pressure
ECOG: Eastern Cooperative Oncology Group
EMR: Electronic Medical Record
EUA: Emergency Use Authorization
HM: Hematologic Malignancies
ICU: Intensive Care Unit
mAbs: Monoclonal Antibodies
O_2_: Oxygen
OTR: Organ Transplant Recipients
SARS-CoV-2: Severe Acute Respiratory Syndrome Coronavirus 2
